# VANL-100 Attenuates Beta-Amyloid-Induced Toxicity in SH-SY5Y Cells

**DOI:** 10.3390/ijms24010442

**Published:** 2022-12-27

**Authors:** Andrila E. Collins, Tarek M. Saleh, Bettina E. Kalisch

**Affiliations:** Department of Biomedical Sciences and Collaborative Specialization in Neuroscience Program, University of Guelph, Guelph, ON N1G 2W1, Canada

**Keywords:** Alzheimer’s disease, beta-amyloid, antioxidants, naringenin, alpha lipoic acid

## Abstract

Antioxidants are being explored as novel therapeutics for the treatment of neurodegenerative diseases such as Alzheimer’s disease (AD) through strategies such as chemically linking antioxidants to synthesize novel co-drugs. The main objective of this study was to assess the cytoprotective effects of the novel antioxidant compound VANL-100 in a cellular model of beta-amyloid (Aβ)-induced toxicity. The cytotoxic effects of Aβ in the presence and absence of all antioxidant compounds were measured using the 3-(4,5-dimethylthiazol-2-yl)2-5-diphenyl-2H-tetrazolium bromide (MTT) assay in SH-SY5Y cells in both pre-treatment and co-treatment experiments. In pre-treatment experiments, VANL-100, or one of its parent compounds, naringenin (NAR), alpha-lipoic acid (ALA), or naringenin + alpha-lipoic acid (NAR + ALA), was administrated 24 h prior to an additional 24-h incubation with 20 μM non-fibril or fibril Aβ_25–35_. Co-treatment experiments consisted of simultaneous treatment with Aβ and antioxidants. Pre-treatment and co-treatment with VANL-100 significantly attenuated Aβ-induced cell death. There were no significant differences between the protective effects of VANL-100, NAR, ALA, and NAR + ALA with either form of Aβ, or in the effect of VANL-100 between 24-h pre-treatment and co-treatment. These results demonstrate that the novel co-drug VANL-100 is capable of eliciting cytoprotective effects against Aβ-induced toxicity.

## 1. Introduction

Alzheimer’s disease (AD) is currently the leading cause of dementia and is projected to affect over 130 million people globally by the year 2050 [1,2,3,4,5]. Despite advancements in research and biomedical technology over the last two decades, its prevalence and incidence rates continue to rise, and the currently available treatment options do not prevent, delay the progression of, or cure AD [6,7,8,9]. In an effort to uncover novel therapies for AD, researchers have begun exploring compounds that target mechanisms implicated in the development and progression of AD. Based on promising pre-clinical findings, human clinical trials began assessing the effectiveness of individual antioxidants or antioxidant combinations that included antioxidant compounds solely or antioxidants combined with drugs currently used to treat AD [10,11,12,13,14,15,16,17,18,19,20,21,22,23,24,25,26,27,28,29,30,31,32]. Antioxidant compounds can be administered together but unbound (combination) or chemically linked (conjugated). The application of antioxidant conjugate therapy (ACT) particularly for AD is based on the role of oxidative stress during the onset and progression of AD. This is outlined in the oxidative stress hypothesis of AD, which postulates the potential mechanisms by which oxidative stress contributes to AD pathology [33,34,35,36,37].

Oxidative stress can be defined as an imbalance between the production and accumulation of toxic reactive oxygen species (ROS) and endogenous antioxidants within the body, resulting in an insufficient or dysfunctional antioxidant defense system [38,39,40,41]. During oxidative stress, ROS target and damage essential cellular structures and biomolecules such as DNA, proteins, and lipids via oxidation [38,39,40,41]. ROS have also been implicated with other pathological hallmarks of AD such as the abnormal aggregation of tau and beta-amyloid (Aβ), further inducing cellular toxicity [42,43,44,45,46,47,48].

Two notable antioxidants that have been explored for their neuroprotective effects in models of neurodegeneration are naringenin (NAR), a naturally occurring polyphenol found in plants and citrus fruit, and alpha-lipoic acid (ALA), an endogenously produced compound that is also found in food such as red meat, broccoli, and spinach [49,50,51,52,53,54]. The individual effects of NAR and ALA in models of AD are well explored in the literature [50,55,56,57,58,59,60,61,62,63,64,65,66,67,68,69,70,71,72,73,74,75,76,77,78,79] and presented as potential therapeutic modalities for targeting and treating AD, as well as functioning as potential chemical constituents for ACT.

**Figure 1:**
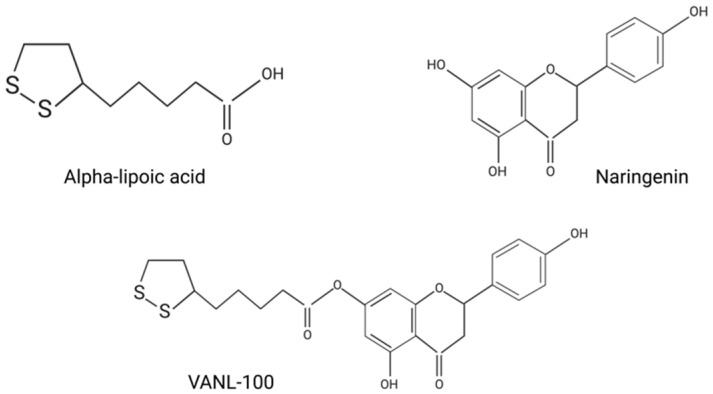
Chemical structures of alpha-lipoic acid (ALA), naringenin (NAR), and VANL-100.

A previous study conducted by Saleh et al. (2017) explored the neuroprotective effects of NAR, ALA, and the novel co-drug VANL-100 (Figure 1), which is the product of the covalent linkage of NAR and ALA. The findings demonstrated the heightened neuroprotective effects of VANL-100, both in vitro by increasing antioxidant capacity and in vivo by reducing infarct volume in a rodent model of an ischemic stroke [80]. Additionally, the novel co-drug VAN-100 was 100 times more potent than the parent compounds, eliciting neuroprotection at lower doses. However, the potential benefits of VANL-100 in other models of neurodegeneration such as AD have not been explored.

In this study, we evaluated the cytoprotective effects of the novel co-drug VANL-100 against Aβ-induced cytotoxicity. Additionally, we compared the effects of VANL-100 to its parent compounds, NAR and ALA, alone and together (NAR + ALA) in a mixture but not covalently bound. This is the first report to explore the effects of NAR and ALA as conjugate therapy in a cellular model of AD. For the purposes of this study, a co-drug is defined as the conjugated drug product from the covalent linkage of two or more chemical entities.

## 2. Results

### 2.1. Non-Fibril and Fibril Aβ_25–35_ Induced Dose-Dependent Cell Death

**Figure 2:**
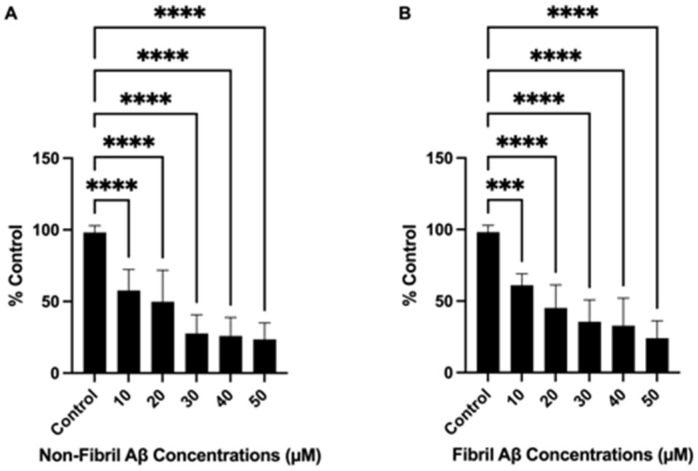
Effects of beta-amyloid (Aβ)_25–35_ on cell viability. Cell viability, represented as % control, is shown on the y-axis, and Aβ_25–35_ concentrations are displayed on the x-axis. The bars represent the mean ± standard error of the mean (SEM) of six independent experiments. Cells were incubated with increasing concentrations (10, 20, 30, 40, and 50 μM) of non-fibril (**A**) or fibril (**B**) Aβ_25–35_. Non-fibril and fibril Aβ_25–35_ reduced cell viability in a dose-dependent manner. Statistical significance vs. controls was assessed using one-way analysis of variance (ANOVA). *** *p* = 0.0001; **** *p* < 0.0001.

Prior to testing the neuroprotective effects of the antioxidant compounds, the toxicities of non-fibril and fibril Aβ_25–35_ were individually tested in SH-SY5Y cells (Figure 2A,B). Considering that the toxicity profile of Aβ differs across various formulations [81,82], we explored the effect of Aβ in its non-fibril and fibrillated states to assess and compare the effects of different compositions on toxicity. Additionally, we utilized the 25–35 protein fragment of Aβ due to its characteristic high toxicity and proven ability to exert neurotoxic effects in AD models [83,84,85]. Since the Aβ_25–35_ peptide is a well-characterized biologically active fragment that retains the toxic properties of Aβ_1–42_, we chose this fragment to assess the potential protective effects of our novel antioxidant and its parent compounds in vitro. Cell viability was evaluated using the 3-(4,5-dimethylthiazol-2-yl)2-5-diphenyl-2H-tetrazolium bromide (MTT) assay after 24 h of Aβ_25–35_ exposure. There were no significant differences in the toxicity levels of non-fibril compared to fibril Aβ_25–35_ in the SH-SY5Y cells in this study. Cell viability was significantly decreased as Aβ concentrations increased compared to untreated controls (*** *p* = 0.0001; **** *p* < 0.0001). We selected 20 μM for both non-fibril and fibril Aβ_25–35_ as the effective dose to carry out subsequent experiments with the antioxidant compounds. This is in line with the literature on effective doses for Aβ treatment to induce cell death in this cell line without completely eradicating all cells [63,86,87,88,89,90,91,92].

### 2.2. Antioxidant Compounds Did Not Induce Cell Loss or Reduce Cell Viability

**Figure 3:**
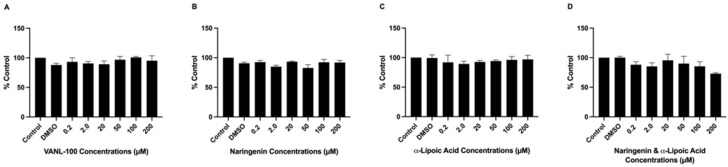
Effects of VANL-100, NAR, ALA, and NAR + ALA on cell viability. Cell viability, represented as % control, is shown on the y-axis, and antioxidant concentrations are displayed on the x-axis. The bars represent the mean ± SEM of three independent experiments. Cells were incubated with different concentrations of each antioxidant and combination (0.2, 2.0, 20, 50, 100 and 200 μM). (**A**) VANL-100; (**B**) NAR alone; (**C**) ALA alone; (**D**) NAR + ALA. None of the antioxidant compounds at any dose significantly induced cell loss or reduced cell viability. Statistical significance vs. controls was assessed using one-way ANOVA. *p* < 0.05.

Figure 3 depicts the effects of VANL-100, NAR, ALA, and NAR + ALA on SH-SY5Y cell viability. None of the antioxidant compounds or combinations resulted in cell loss at 0.2, 2.0, 20, 50, 100, or 200 μM (*p* > 0.05). Cell viability was evaluated using the MTT assay 24 h after exposure to the antioxidant compounds. This assessment was necessary to confirm that the antioxidant compounds were not exerting toxic effects on the cells alone, prior to treatment with Aβ.

### 2.3. Effect of Antioxidant Compounds on Aβ-Induced Cell Death

**Figure 4:**
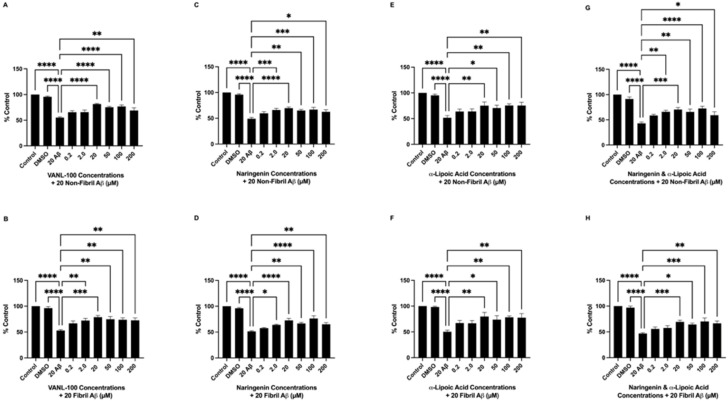
Viability of SH-SY5Y cells pre-treated with VANL-100, NAR, ALA, or NAR + ALA for 24 h followed by 24-h treatment with Aβ_25–35_. Cell viability, represented as % control, is shown on the y-axis, and antioxidant concentrations are displayed on the x-axis. The bars represent the mean ± SEM of six independent experiments. (**A**,**B**) VANL-100 + non-fibril and fibril Aβ_25–35_, respectively. (**C**,**D**) NAR + non-fibril and fibril Aβ_25–35_, respectively. (**E**,**F**) ALA + non-fibril and fibril Aβ_25–35_, respectively. (**G**,**H**) NAR + ALA + non-fibril and fibril Aβ_25–35_, respectively. Statistical significance vs. Aβ was assessed using one-way ANOVA. * *p* <0.05; ** *p*< 0.01; *** *p* < 0.001; **** *p* < 0.0001.

In 24-h pre-treatment and co-treatment experiments, the effect of VANL-100, NAR, ALA, and NAR + ALA were assessed against non-fibril and fibril Aβ_25–35_ in SH-SY5Y cells. During pre-treatment, cells were treated with 0.2, 2.0, 20, 50, 100, or 200 μM of each antioxidant 24 h prior to Aβ_25–35_ addition. After 24 h, cells were co-incubated with the same doses of the antioxidants in addition to 20 μM non-fibril or fibril Aβ_25–35_, which was followed by cell viability measurement via MTT assay 24 h later. In co-treatment, cells received 0.2, 2.0, 20, 50, 100, or 200 μM of each antioxidant simultaneously with 20 μM non-fibril and fibril Aβ_25–35_, and cell viability was measured 24 h later via MTT assay. Results from pre-treatment experiments (Figure 4A–H) revealed significant improvements in cell viability for all antioxidant compounds when combined with Aβ_25–35_ compared to treatment with Aβ_25–35_ alone. VANL-100 significantly increased cell viability compared to non-fibril Aβ_25–35_ alone at doses of 20, 50, 100 (*p* < 0.0001), and 200 μM (*p* = 0.0066) and fibril Aβ_25–35_ at doses of 2.0 (*p* = 0.0051), 20 (*p* = 0.0001), 50 (*p* = 0.0013), 100 (*p* = 0.0022) and 200 μM (*p* = 0.0041), as shown in Figure 4A,B, respectively. NAR significantly increased cell viability compared to non-fibril Aβ_25–35_ at 2.0 (*p* = 0.0008), 20 (*p* < 0.0001), 50 (*p* = 0017), 100 (*p* = 0.0004) and 200 μM (*p* = 0.0104), and fibril Aβ_25–35_ at doss of 2.0 (*p* = 0.0139), 20 (*p* < 0.0001), 50 (*p* = 0018), 100 (*p* < 0.0001), 200 μM (*p* = 0.0061), as shown in Figure 4C,D, respectively. ALA significantly attenuated the reduction in cell viability induced by non-fibril Aβ_25–35_ at doses of 20 (*p* = 0.0070), 50 (*p* = 0.0451), 100 (*p* = 0.0058), 200 μM (*p* = 0.0063), and fibril Aβ_25–35_ at doses of 20 (*p* = 0.0025), 50 (*p* = 0.0230), 100 (*p* = 0.0045), 200 μM (*p* = 0.0059) as shown in Figure 4E,F, respectively. The combination of NAR + ALA also significantly increased cell viability compared to non-fibril Aβ_25–35_ alone at doses of 2.0 (*p* = 0.0018), 20 (*p* = 0.0002), 50 (*p* = 0020), 100 (*p* = 0.0001), 200 μM (*p* = 0.0423), and fibril Aβ_25–35_ at doses of 20 (*p* = 0.0008), 50 (*p* = 0129), 100 (*p* = 0.0005), 200 μM (*p* = 0.0044), as shown in Figure 4G,H, respectively.

**Figure 5:**
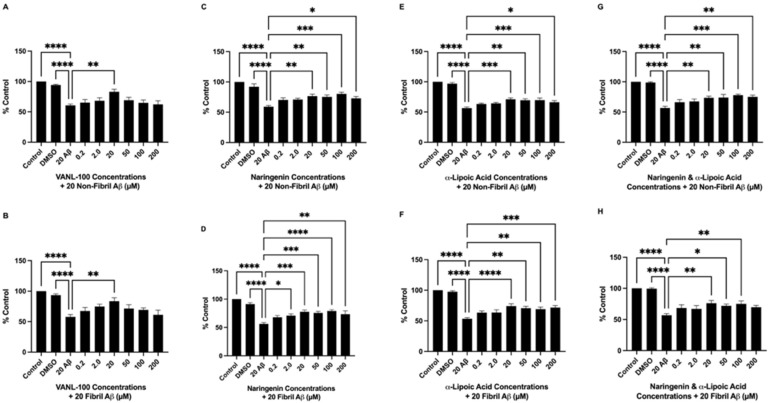
Viability of SH-SY5Y cells co-treated with VANL-100, NAR, ALA, or NAR + ALA and Aβ_25–35_ for 24 h. Cell viability, represented as % control, is shown on the y-axis, and antioxidant concentrations are displayed on the x-axis. The bars represent the mean ± SEM of six independent experiments. (**A**,**B**) VANL-100 + non-fibril and fibril Aβ_25–35_, respectively. (**C**,**D**) NAR + non-fibril and fibril Aβ_25–35_, respectively. (**E**,**F**) ALA + non-fibril and fibril Aβ_25–35_, respectively. (**G**,**H**) NAR + ALA + non-fibril and fibril Aβ_25–35_, respectively. Statistical significance vs. Aβ was assessed using one-way ANOVA. * *p* <0.05; ** *p*< 0.01; *** *p* < 0.001; **** *p* < 0.0001.

Similarly, results from co-treatment experiments (Figure 5A–H) indicated significant improvement in viability when cells were treated with Aβ_25–35_ in combination with each of the antioxidant compounds compared to treatment with Aβ_25–35_ alone. Co-treatment with VANL-100 significantly improved cell viability compared to non-fibril Aβ_25–35_ at 20 μM (*p* = 0.0017) and fibril Aβ_25–35_ at 20 μM (*p* = 0.0019), as shown in Figure 5A,B, respectively. NAR co-treatment significantly increased cell viability compared to non-fibril Aβ_25–35_ at 20 (*p* = 0.0014), 50 (*p* = 0038), 100 (*p* = 0.0001), and 200 μM (*p* = 0.0161), and fibril Aβ_25–35_ at 2.0 (*p* = 0.0115), 20 (*p* = 0.0001), 50 (*p* = 0.0005), 100 (*p* < 0.0001), and 200 μM (*p* = 0.0023), as shown in Figure 5C,D, respectively. ALA co-treatment significantly increased cell viability compared to non-fibril Aβ_25–35_ at 20 (*p* = 0.0003), 50 (*p* = 0.0011), 100 (*p* = 0.0010), and 200 μM (*p* = 0.0207), and fibril Aβ_25–35_ at 20 (*p* < 0.0001), 50 (*p* = 0.0011), 100 (*p* = 0.0031), and 200 μM (*p* = 0.0005), as shown in Figure 5E,F, respectively. Co-treatment with the combination of NAR + ALA significantly improved cell viability compared to non-fibril Aβ_25–35_ at 20 (*p* = 0.034), 50 (*p* = 0.0026), 100 (*p* = 0.0002), 200 μM (*p* = 0.0012), and fibril Aβ_25–35_ at 20 (*p* = 0.0036), 50 (*p* = 0.0338), and 100 (*p* = 0.0066), as shown in Figure 5G,H, respectively. A summary of these results is shown in Table 1.

### 2.4. Cytoprotective Effects of VANL-100 Are Not Significantly Different to Parent Compounds

**Figure 6:**
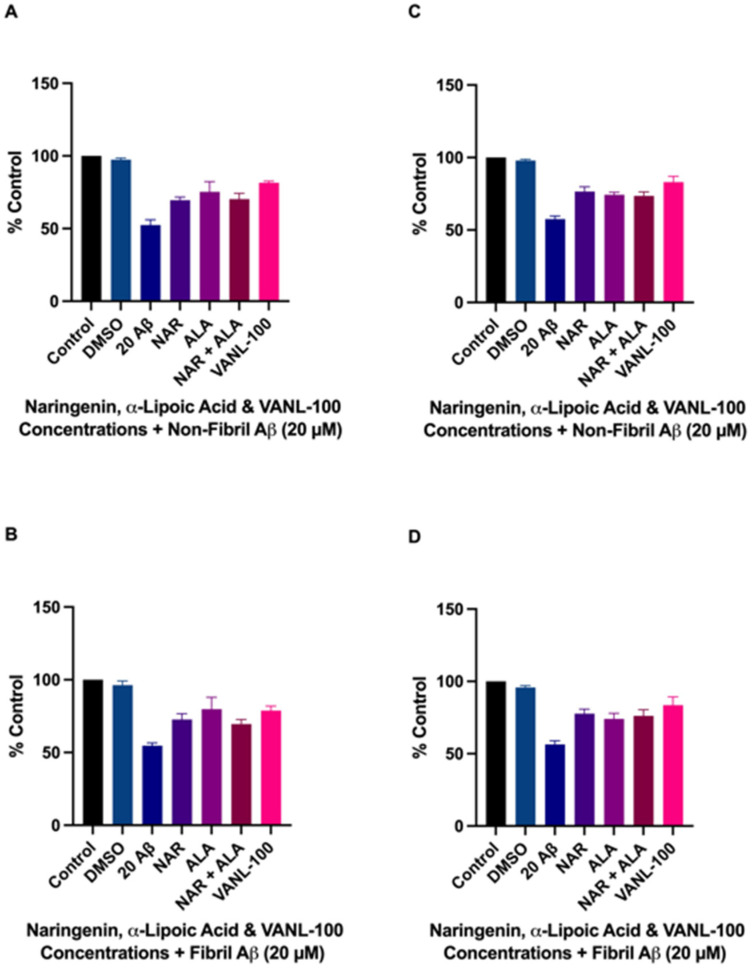
Effects of pre-treatment and co-treatment with VANL-100 compared to parent compounds. Cell viability, represented as % control, is shown on the y-axis, and antioxidant concentrations are displayed on the x-axis. The bars represent the mean ± SEM of six independent experiments. (**A**,**B**) Pre-treatment with VANL-100 compared to parent compounds against non-fibril and fibril Aβ_25–35_, respectively. (**C**,**D**) Co-treatment with VANL-100 compared to parent compounds against non-fibril and fibril Aβ_25–35_, respectively. Statistical significance vs. VANL-100 was assessed using one-way ANOVA. *p* < 0.05.

Of the three doses (20, 50, and 100 μM) that elicited the greatest neuroprotective effects on cell survival, we selected the 20 μM dose to compare the protective effects of VANL-100 to NAR, ALA, and NAR + ALA to determine if VANL-100 elicits greater protective effects than its parent compounds. Comparisons were made for both pre-treated and co-treated cells in combination with either 20 μM non-fibril or fibril Aβ_25–35_. For both non-fibril and fibril Aβ_25–35_ (Figure 6A,B, respectively), the cytoprotective effects of cells pre-treated with VANL-100 were not significantly different from those observed in cells pre-treated with 20 μM NAR, ALA, or NAR + ALA. Similarly, following co-treatment with non-fibril and fibril Aβ_25–35_ (Figure 6C,D, respectively), the cytoprotective effects of VANL-100 were not significantly different (*p* > 0.05) from its parent compounds alone or when combined but not covalently linked.

### 2.5. Effect of Treatment Timepoint on Cell Survival

**Figure 7:**
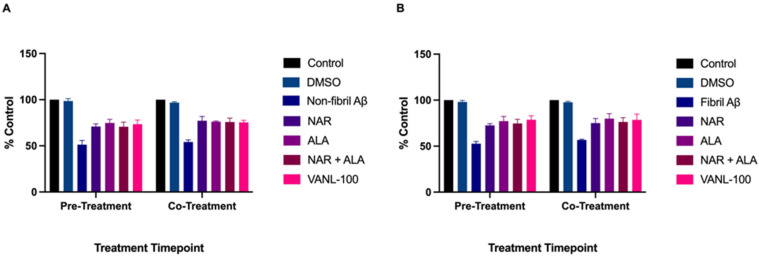
Effect of antioxidant treatment timepoint on overall cell survival. Cell viability, represented as % control, is shown on the y-axis, and antioxidant treatment time is displayed on the x-axis. The bars represent the mean ± SEM of three independent experiments. (**A**) Comparison of pre-treatment and co-treatment of antioxidants in combination with non-fibril Aβ_25–35_. (**B**) Comparison of pre-treatment and co-treatment of antioxidants in combination with fibril Aβ_25–35_. Statistical significance of pre-treatment vs. co-treatment was assessed using two-way ANOVA. *p* < 0.05.

Finally, we evaluated the overall protective effects of 24-h pre-treatment compared to co-treatment with 20 μM VANL-100, NAR, ALA, or NAR + ALA against 20 μM non-fibril and fibril Aβ_25–35_ (Figure 7). Although all antioxidant compounds elicited cytoprotection and increased cell survival in non-fibril and fibril Aβ_25–35_ treated cells, there was no significant difference in cell survival between the overall effect of 24-h pre-treatment and co-treatment (*p* > 0.05) for VANL-100, NAR, ALA, or NAR + ALA.

### 2.6. Effect of VANL-100 on ROS Levels in SH-SY5Y Cells

**Figure 8:**
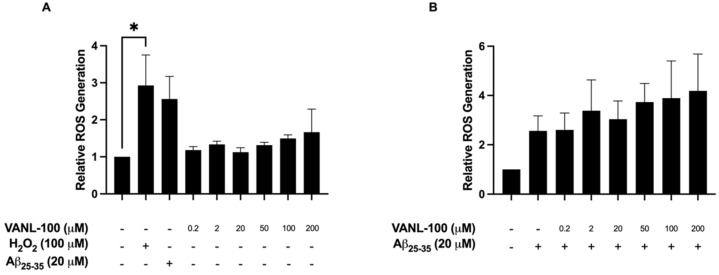
Effect of VANL-100 treatment on ROS generation in SH-SY5Y cells. Relative ROS generation is shown on the y-axis, and individual treatments are displayed on the x-axis. The bars represent the mean ± SEM of four independent experiments. (**A**) Effect of H_2_O_2_, Aβ_25–35_, and VANL-100 alone on ROS production. (**B**) Effect of Aβ_25–35_ alone and VANL-100 with Aβ_25–35_ on ROS production. Statistical significance was assessed using one-way ANOVA. * *p* < 0.05.

To determine the generation of intracellular ROS induced by Aβ_25–35_, we utilized the 2′,7′-dichllorodihydrofluorescein diacetate (H_2_DCFDA) fluorescence assay. SH-SY5Y cells were treated with 20 μM fibril Aβ_25–35_, as well as increasing doses of VANL-100 either alone or co-treated with 20 μM fibril Aβ_25–35._ Cells were treated with 100 μM hydrogen peroxide (H_2_O_2_) as a positive control, and the H_2_DCFDA assay results showed that treatment with H_2_O_2_ significantly increased ROS generation (*p* = 0.0149; Figure 8A). Although relative ROS levels were consistently higher in cells treated with Aβ_25–35_ alone (Figure 8A), this increase was not statistically different from control ROS levels. Treatment with increasing doses of VANL-100 alone (0.2, 2, 20, 50, 100, and 200 μM) did not significantly increase ROS production (*p* > 0.05; Figure 8A), supporting our viability results that VANL-100 does not induce cellular toxicity when administered alone. The level of ROS following co-treatment with VANL-100 and 20 μM fibril Aβ_25–35_ was not significantly different from Aβ_25–35_ exposure alone (Figure 8B); however, values were also not statistically different from those detected in control cells. These results indicate that VANL-100 alone does not induce ROS production, but its ability to scavenge ROS may be impaired by the presence of Aβ_25–35_.

## 3. Discussion

The objectives of this study were to assess the neuroprotective capability of the novel antioxidant co-drug VANL-100 in an in vitro model of AD and to determine whether its cytoprotective effects were greater than its parent compounds, NAR and ALA, either alone or combined but not covalently linked (NAR + ALA). Additionally, we aimed to explore the applicability of VANL-100 as a form of ACT for the prevention (pre-treatment) or treatment of neurotoxicity associated with AD pathology. We first assessed the neurotoxicity induced by various doses of both non-fibril and fibril Aβ_25–35_. The results indicated a dose-dependent decrease in SH-SY5Y cell viability as Aβ concentrations increased. We then selected the 20 μM dose as the treatment of non-fibril and fibril Aβ_25–35_ to be applied in subsequent antioxidant experiments, as this dose induced cell death but did not eradicate all cells. This is in line with the current literature, which identifies this as an appropriate dose for assessing Aβ_25–35_-induced cell death, particularly in the SH-SY5Y cell line [63,86,87,88,89,90,91,92]. We then assessed whether the antioxidant compounds VANL-100, NAR, ALA, or NAR + ALA exerted any toxic effects on the cells. Results indicated that these compounds did not reduce cell viability or induce cell death. Overall, our results indicate that the novel conjugate drug VANL-100 can provide cytoprotection against Aβ-induced cell loss. This protection was observed at various doses (20, 50, 100, and 200 μM) and both when cells were pre-treated 24 h prior to Aβ exposure and when co-treated with Aβ. Additionally, both parent compounds, NAR and ALA, when alone and when combined but not covalently linked (NAR + ALA), also attenuated the reduction in cell viability induced by Aβ at similar concentrations (2.0, 20, 50, 100, and 200 μM), during both pre-treatment and co-treatment. For all treatment conditions, there were no significant differences in the neuroprotective effects of VANL-100, NAR, ALA, or NAR + ALA and no significant difference in the cytoprotective effects elicited by the antioxidant compounds following either 24-h pre-treatment or co-treatment.

The involvement of oxidative stress in AD has been validated, and our findings provide early evidence of the potential use of antioxidants, alone and in combined or conjugated forms, to address Aβ toxicity. Since the currently available treatment options for AD such as donepezil, rivastigmine, and memantine [7,93,94] are ineffective in preventing, reversing, or slowing the progression of the disease and are primarily indicated for symptom management, it is necessary to explore alternative methods for preventing and treating AD. More recently, researchers have explored disease-modifying candidate compounds and drugs [8,95]. However, they have proven ineffective, likely due to an insufficient understanding of the intricate pathophysiology of AD and an inappropriate selection of treatment targets and dosages. Addressing the presence of oxidative stress in AD pathology via antioxidants and ACT serves as a promising approach to mitigating oxidative damage and targeting the interaction between ROS and Aβ that contributes to the onset and development of AD.

Researchers have reported the role of Aβ-induced oxidative stress through mechanisms that include the presence of extracellular senile plaques/fibrils comprised of aggregated Aβ peptides with the metal ions iron, copper, and zinc [96,97,98]. These redox-active metals can catalyze the production of ROS when bound to Aβ [96,97,98]. Subsequently, newly generated ROS can oxidize both Aβ peptides as well as surrounding biomolecules. The oxidation of biomolecules such as lipids within neuronal membranes obstructs membrane integrity [99]. Due to its oxidation by ROS and redox-active metals, Aβ clearance is impaired and significantly decreased in those with AD [42,100]. In turn, Aβ exerts toxic effects through the dysregulation of calcium homeostasis and membrane potential depletion, disrupting the cytoskeleton and synaptic function which stimulates neuronal apoptosis [101,102,103,104,105,106]. This has led researchers to explore antioxidant-based targets to address the involvement of oxidative stress in AD pathology.

The improvement in cellular metabolism observed in cells treated with Aβ_25–35_ and the antioxidant compounds (VANL-100, NAR, or ALA) compared to cells treated with Aβ_25–35_ alone can be attributed to various factors including the pathways proposed to be involved in Aβ-induced cell death and the ability of the current antioxidant compounds to attenuate the activation of these pathways. It is reported in the literature that Aβ induces neuronal death via apoptotic mechanisms such as c-Jun N-terminal kinase (JNK) activation, caspase-3 activation, and p38 mitogen-activated protein kinase (MAPK) activation, including neuroinflammatory pathways such as nuclear factor kappa B (NFκB) [107,108,109,110,111,112,113]. These pathways have also been implicated in oxidative stress-induced cell death, and the utilization of compounds that stimulate the production of antioxidant and detoxifying enzymes has been shown to inhibit apoptosis, resulting in reduced cell death [114,115,116]. Our current results are in line with the literature and support the neuroprotective role of antioxidants NAR and ALA against Aβ-induced toxicity.

**Figure 9:**
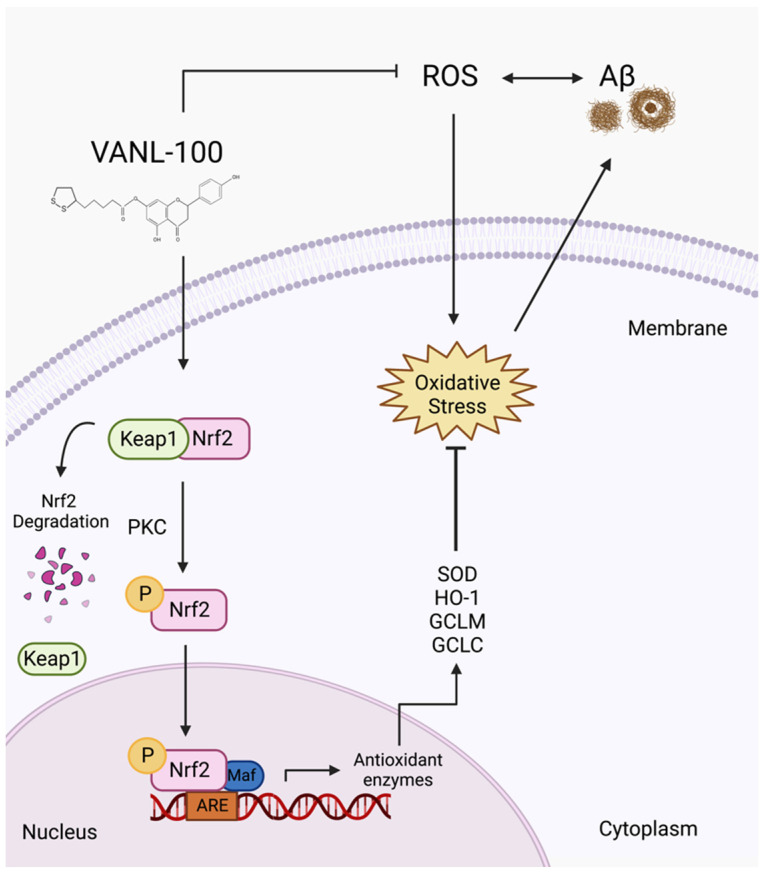
Schematic of the proposed mechanism of action of VANL-100. VANL-100 may act as an Nrf2 activator to stimulate the production of antioxidant and detoxifying enzymes that inhibit oxidative stress and its contribution to the production and accumulation of Aβ. Created using BioRender.com.

Interestingly, both NAR and ALA have been reported to elicit their neuroprotective antioxidant effects through similar mechanisms and using similar intracellular antioxidant signaling pathways, including through the activation of nuclear factor erythroid 2-related factor 2 (Nrf2) [117,118,119,120,121,122,123,124,125,126,127,128]. Nrf2 is the primary transcription factor responsible for the activity of the antioxidant defense system; it regulates the expression of antioxidant and detoxifying genes and the subsequent production of antioxidant and detoxifying enzymes [129,130,131,132]. Nrf2 may serve as a potential mechanism of protection for VANL-100 (Figure 9). We propose that VANL-100 activates Nrf2 by stimulating the cytoplasmic dissociation of Nrf2 from the inhibitory protein Kelch-like ECH-associated protein 1 (Keap1) via phosphorylation by kinases such as PKC. Activation of Nrf2 leads to the nuclear translocation of phosphorylated Nrf2, where Nrf2 binds to small Maf proteins on the antioxidant response element (ARE) in the promoter region of antioxidant genes such as superoxide dismutase (SOD), heme-oxygenase 1 (HO-1), glutamate-cysteine ligase modifiers and catalytic subunits (GCLM and GCLC, respectively). This stimulates the transcription of antioxidant and detoxifying genes that code for enzymes that inhibit oxidative stress and the subsequent production of Aβ plaques.

Our results demonstrated the cytoprotective effects of all antioxidant compounds studied, but in contrast to the findings of Saleh et al. (2017), we did not see enhanced protection from VANL-100 compared to NAR and ALA when alone or in combination but not covalently bound (NAR + ALA). There are several factors that may have contributed to these findings. It is important to recognize that Saleh et al. (2017) tested the effects of VANL-100 and its parent compounds in a rodent model of an ischemic stroke. Since the pathophysiologies of an ischemic stroke and AD are significantly different, and the complexity of in vivo neurotoxicity is not always captured in vitro, it is expected that discrepancies in our findings may exist when utilizing two different models of neurodegeneration. Additionally, Saleh et al. (2017) used oxygen and glucose deprivation in mixed neocortical primary cultures for their in vitro experiments, whereas we used SH-SY5Y cells treated with Aβ in our cellular model. It is plausible that, compared to its parent compounds, the effects of this conjugated drug, VANL-100, may have a distinctive impact based on the mechanisms of neurotoxicity and/or in neuronal cells compared to the neuroblastoma cell line. Taken together, these factors likely contributed to the observation that the neuroprotective effects of VANL-100 in the present study were not significantly different from those of the parent compounds.

Researchers have reported increased ROS production following Aβ exposure in various cell lines including SH-SY5Y cells [133,134,135,136,137,138,139,140]. Additionally, antioxidant compounds such as NAR and ALA have been shown to attenuate cell stress due to elevated ROS production induced by Aβ exposure and other mediators of oxidative stress [64,68,76,118,141,142]. Since the neuroprotective effects of the parent compounds NAR and ALA against ROS generation have already been described in the literature, it was necessary to also assess whether the novel compound VANL-100 exerts similar effects on ROS generation in vitro. H_2_O_2_ was used as a positive control and significantly increased ROS generation. When administered alone, VANL-100 did not significantly increase ROS production, which supports results from cell viability experiments that indicate that VANL-100 does not exert toxic effects on SH-SY5Y cells. Although not statistically significant, Aβ_25–35_ treatment alone consistently resulted in higher ROS levels which remained elevated when cells were co-treated with Aβ_25–35_ and VANL-100. It is possible that Aβ_25–35_ may be inducing an antioxidant response that masks some of the effects of Aβ_25–35_ on ROS levels. Future studies should examine ROS target molecules to gain a better understanding of the mechanism of Aβ_25–35_-induced cell death in SH-SY5Y cells. The results also suggest that VANL-100 is not protecting SH-SY5Y cells via the inhibition of ROS generation. While this is possible, additional experiments would be needed to better understand the mechanisms involved. It is possible that the toxic effects of Aβ_25–35_-induced ROS generation may interfere with the capacity of VANL-100 to scavenge ROS. Additionally, the variability in the data, along with the lack of significant increase in ROS following Aβ_25–35_ treatment, indicates that additional optimization of the assay or alternative methods for examining ROS should be carried out. Although significant improvements in viability were observed following 24 h of co-treatment with Aβ_25–35_ and VANL-100, this timepoint may not be optimal for assessing ROS levels. Reproducing these experiments across various time points may provide evidence for the impact of VANL-100 on ROS levels. Additional experiments, including the examination of ROS targets, are necessary to discern the neuroprotective effects VANL-100 exerts on reducing intracellular ROS production in this cell line.

The SH-SY5Y cell line is widely utilized in studies requiring a reliable neuron-like cell culture model, which supports our use of SH-SY5Y in this study. However, the utilization of this neuroblastoma cell line is also the main limitation of our study. It is arguable that the cell line may not be the most appropriate model of AD since it is a cancer cell line. The SH-SY5Y cell line can be further manipulated using agents such as retinoic acid to stimulate differentiation and morphological characteristics comparable to neuronal cells [143,144]. Additionally, an alternative method for modelling AD could include the utilization of primary neurons with genetic modifications that mimic AD. These include, but are not limited to, neurons derived from single, double, and triple transgenic AD mouse models, which are frequently utilized in preclinical studies [145]. Utilizing animal models that meticulously recapitulate some of the clinical pathologies of AD are necessary next steps for discerning molecular mechanisms and advancing preclinical investigations of ACTs.

Future directions include exploring the mechanisms by which VANL-100 elicits neuroprotective effects in the SH-SY5Y cellular model of AD. As previously mentioned, the parent compounds of VANL-100, NAR, and ALA, possess antioxidant neuroprotective effects through their mechanism of action as activators of Nrf2, the primary transcription factor involved in the body’s endogenous antioxidant response to oxidative stress [117,118,119,120,121,122,123,124,125,126,127,128]. It is plausible that VANL-100 may provide neuroprotection by acting through similar mechanisms as NAR and ALA to activate Nrf2 signaling pathways and stimulate the production of antioxidant and detoxifying enzymes that target perpetrators of oxidative stress such as ROS. Additionally, it is necessary to assess the effects of VANL-100 in other models of AD, including, but not limited to, primary neurons, including those obtained from transgenic AD mice and in vivo explorations, since researchers have reported the neuroprotective effects of its parent compounds, NAR and ALA, in transgenic and non-transgenic AD rodent models [58,66,72,73,146,147,148,149]. Although both parent compounds of VANL-100 have been shown to provide neuroprotective effects in various models of AD, our work is the first to explore the role of VANL-100 as a form of ACT in an early cellular model of AD.

## 4. Materials and Methods

### 4.1. Materials

VANL-100 was synthesized following the synthesis and proton NMR validation protocol previously described [80]. NAR was obtained from Sigma-Aldrich (Cat. No. N5893). ALA was obtained from EMD Millipore (Cat. No. 437692). VANL-100, NAR, and ALA were dissolved in DMSO (Sigma Life Science, Cat. No. D2650). Aβ_25–35_ was purchased from Cedarlane (product No. A15416-25) and prepared in sterile distilled water at a concentration of 1 mM. Aliquots were stored at −20 °C until treatment. For experiments examining the effects of soluble Aβ_25–35,_ aliquots were used immediately after thawing, to limit aggregation of the peptide. Aggregates were prepared as described previously, with aliquots incubated at 37 °C for four days prior to treatment [150]. MTT was purchased from EMD Millipore (Cat. No. 475989) and dissolved in phosphate buffer saline (PBS). H_2_DCFDA was purchased from Invitrogen by Thermo Fisher Scientific (Cat. No. C6827) and dissolved in DMSO.

### 4.2. Cell Culture

The human neuroblastoma SH-SY5Y cell line was obtained from ATCC (CRL-2266) and maintained in Dulbecco’s Modified Eagle Medium (DMEM; Gibco by Life Technologies) supplemented with 10% fetal bovine serum (FBS; Gibco by Life Technologies) and 5% penicillin-streptomycin (Gibco by Life Technologies). The cells were grown and maintained at 37 °C in a humidified environment of 5% CO_2_ and 95% air.

### 4.3. Antioxidant Treatments

SH-SY5Y cells were seeded in 96-well plates and treated with antioxidants once they reached 70–80% confluence. Pre-treatment experiments consisted of 24-h treatment with 0.2, 2.0, 20, 50, 100, and 200 μM of VANL-100, NAR alone, ALA alone, or NAR + ALA, followed by co-incubation with 20 μM non-fibril or fibril Aβ_25–35_ for an additional 24 h. Co-treatment experiments consisted of simultaneous co-treatment with 0.2, 2.0, 20, 50, 100, and 200 μM of each antioxidant compound with 20 μM non-fibril or fibril Aβ_25–35_. Twenty-four hours after Aβ_25–35_ administration, cell viability was assessed using the MTT assay. DMSO was used to dissolve the antioxidants and was therefore used as the vehicle in all experiments. The final concentration of DMSO in all wells was 0.1%.

### 4.4. Cell Viability and Toxicity Assay

After 24 h of incubation, the media was aspirated and 100 μL of the MTT reagent (0.75 mg/mL in PBS) was added to each well and incubated for 3 h. Post incubation, the MTT reagent was removed and 100 μL of DMSO was added to each well. Following incubation for 15 min, absorbance was read at 600 nm. The cell viability and toxicity of all antioxidant compounds and Aβ_25–35_ were individually established in addition to the protective effects of VANL-100 and its parent compounds against non-fibril or fibril Aβ_25–35_-treated cells.

### 4.5. Reactive Oxygen Species Determination

ROS levels were detected by the H_2_DCFDA fluorescence assay [151]. Cells were seeded in black clear-bottom 96-well plates in phenol-red free media and incubated overnight. On the day of the experiment, 20 μM H_2_DCFDA was prepared in phenol-red free media, 100 μL was added to each well, and the plates were incubated for 30 min. The media was aspirated, and the cells were fed with fresh phenol-red free media. Cells were then treated with antioxidant compounds and Aβ_25–35_. After 24 h of incubation, ROS generation was measured with fluorescence plate spectroscopy at an excitation of 485 nm and an emission of 520 nm. Cells treated with 100 μM H_2_O_2_ served as a positive control. Because cell viability was reduced following 24-h treatment with Aβ_25–35_, MTT assay measurements from the same wells were used to normalize the ROS values.

### 4.6. Statistical Analysis

All data are represented as a mean ± standard error of the mean (SEM). Statistical analyses were performed and interpreted using GraphPad Prism 9. One-way analysis of variance (ANOVA) was conducted to compare control groups and treatment groups, followed by Dunnett’s post-hoc test. Two-way ANOVA was conducted to compare the effects of pre-treatment and co-treatment time points, followed by Sidak’s post-hoc test. Statistical significance was defined by *p* < 0.05.

## 5. Conclusions

Our results demonstrate for the first time the neuroprotective effects of the novel co-drug VANL-100 in an Aβ-induced cellular model of AD. These findings support the use of antioxidant combinations or conjugate compounds as a potential novel therapeutic strategy for combating oxidative stress and the impact of abnormal protein aggregation during the onset and progression of AD. Additionally, our results support the role of oxidative stress in the pathogenesis of AD and the necessary presence of antioxidants to maintain optimal cellular conditions and overall cell health. Future studies exploring the mechanisms by which VANL-100 elicits neuroprotection are necessary to elucidate the underlying molecular pathways involved in its protection of and applicability to other models/conditions of neurodegeneration. This would further elucidate the efficacy and appropriateness of VANL-100 and other antioxidant-based co-drugs as potential drug candidates for the prevention and/or treatment of neurodegenerative diseases that are characterized by oxidative stress and/or abnormal protein aggregation.

## Figures and Tables

**Table 1:** Summary of results. NAR: Naringenin; ALA: Alpha-lipoic acid; NAR + ALA; Naringenin + Alpha-lipoic acid; NF: Non-fibril; F: Fibril.

Timepoint	Antioxidant Compound(s)	Treatment	Result (*p*-Value)	Figure
24-h pre-treatment	VANL-100	0.2 μM + 20 μM NF Aβ 0.2 μM + 20 μM F Aβ	*p* > 0.05 *p* > 0.05	Figure 4A,B
		2.0 μM + 20 μM NF Aβ 2.0 μM + 20 μM F Aβ	*p* > 0.05 *p* = 0.0051	
		20 μM + 20 μM NF Aβ 20 μM + 20 μM F Aβ	*p* < 0.0001 *p* = 0.0001	
		50 μM + 20 μM NF Aβ 50 μM + 20 μM F Aβ	*p* < 0.0001 *p* = 0.0013	
		100 μM + 20 μM NF Aβ 100 μM + 20 μM F Aβ	*p* < 0.0001 *p* = 0.0022	
		200 μM + 20 μM NF Aβ 200 μM + 20 μM F Aβ	*p* = 0.0066 *p* = 0.0041	
	NAR	0.2 μM + 20 μM NF Aβ 0.2 μM + 20 μM F Aβ	*p* > 0.05 *p* > 0.05	Figure 4C,D
		2.0 μM + 20 μM NF Aβ 2.0 μM + 20 μM F Aβ	*p* = 0.0008 *p* = 0.0139	
		20 μM + 20 μM NF Aβ 20 μM + 20 μM F Aβ	*p* < 0.0001 *p* < 0.0001	
		50 μM + 20 μM NF Aβ 50 μM + 20 μM F Aβ	*p* = 0.017 *p* = 0.0018	
		100 μM + 20 μM NF Aβ 100 μM + 20 μM F Aβ	*p* = 0.0004 *p* < 0.0001	
		200 μM + 20 μM NF Aβ 200 μM + 20 μM F Aβ	*p* = 0.0104 *p* = 0.0061	
	ALA	0.2 μM + 20 μM NF Aβ 0.2 μM + 20 μM F Aβ	*p* > 0.05 *p* > 0.05	Figure 4E,F
		2.0 μM + 20 μM NF Aβ 2.0 μM + 20 μM F Aβ	*p* > 0.05 *p* > 0.05	
		20 μM + 20 μM NF Aβ 20 μM + 20 μM F Aβ	*p* = 0.0070 *p* = 0.0025	
		50 μM + 20 μM NF Aβ 50 μM + 20 μM F Aβ	*p* = 0.0451 *p* = 0.0230	
		100 μM + 20 μM NF Aβ 100 μM + 20 μM F Aβ	*p* = 0.0058 *p* = 0.0045	
		200 μM + 20 μM NF Aβ 200 μM + 20 μM F Aβ	*p* = 0.0063 *p* = 0.0059	
	NAR + ALA	0.2 μM + 20 μM NF Aβ 0.2 μM + 20 μM F Aβ	*p* > 0.05 *p* > 0.05	Figure 4G,H
		2.0 μM + 20 μM NF Aβ 2.0 μM + 20 μM F Aβ	*p* = 0.0018 *p* > 0.05	
		20 μM + 20 μM NF Aβ 20 μM + 20 μM F Aβ	*p* = 0.0002 *p* = 0.0008	
		50 μM + 20 μM NF Aβ 50 μM + 20 μM F Aβ	*p* = 0.0020 *p* = 0.0129	
		100 μM + 20 μM NF Aβ 100 μM + 20 μM F Aβ	*p* = 0.0001 *p* = 0.0005	
		200 μM + 20 μM NF Aβ 200 μM + 20 μM F Aβ	*p* = 0.0423 *p* = 0.0044	
Co-treatment	VANL-100	0.2 μM + 20 μM NF Aβ 0.2 μM + 20 μM F Aβ	*p* > 0.05 *p* > 0.05	Figure 5A,B
		2.0 μM + 20 μM NF Aβ 2.0 μM + 20 μM F Aβ	*p* > 0.05 *p* > 0.05	
		20 μM + 20 μM NF Aβ 20 μM + 20 μM F Aβ	*p* = 0.0017 *p* = 0.0019	
		50 μM + 20 μM NF Aβ 50 μM + 20 μM F Aβ	*p* > 0.05 *p* > 0.05	
		100 μM + 20 μM NF Aβ 100 μM + 20 μM F Aβ	*p* > 0.05 *p* > 0.05	
		200 μM + 20 μM NF Aβ 200 μM + 20 μM F Aβ	*p* > 0.05 *p* > 0.05	
	NAR	0.2 μM + 20 μM NF Aβ 0.2 μM + 20 μM F Aβ	*p* > 0.05 *p* > 0.05	Figure 5C,D
		2.0 μM + 20 μM NF Aβ 2.0 μM + 20 μM F Aβ	*p* > 0.05 *p* = 0.0115	
		20 μM + 20 μM NF Aβ 20 μM + 20 μM F Aβ	*p* = 0.0014 *p* = 0.0001	
		50 μM + 20 μM NF Aβ 50 μM + 20 μM F Aβ	*p* = 0.0038 *p* = 0.0005	
		100 μM + 20 μM NF Aβ 100 μM + 20 μM F Aβ	*p* < 0.0001 *p* = 0.0001	
		200 μM + 20 μM NF Aβ 200 μM + 20 μM F Aβ	*p* = 0.0161 *p* = 0.0023	
	ALA	0.2 μM + 20 μM NF Aβ 0.2 μM + 20 μM F Aβ	*p* > 0.05 *p* > 0.05	Figure 5E,F
		2.0 μM + 20 μM NF Aβ 2.0 μM + 20 μM F Aβ	*p* > 0.05 *p* > 0.05	
		20 μM + 20 μM NF Aβ 20 μM + 20 μM F Aβ	*p* = 0.0003 *p* < 0.0001	
		50 μM + 20 μM NF Aβ 50 μM + 20 μM F Aβ	*p* = 0.0011 *p* = 0.0011	
		100 μM + 20 μM NF Aβ 100 μM + 20 μM F Aβ	*p* = 0.0010 *p* = 0.0031	
		200 μM + 20 μM NF Aβ 200 μM + 20 μM F Aβ	*p* = 0.0207 *p* = 0.0005	
	NAR + ALA	0.2 μM + 20 μM NF Aβ 0.2 μM + 20 μM F Aβ	*p* > 0.05 *p* > 0.05	Figure 5G,H
		2.0 μM + 20 μM NF Aβ 2.0 μM + 20 μM F Aβ	*p* > 0.05 *p* > 0.05	
		20 μM + 20 μM NF Aβ 20 μM + 20 μM F Aβ	*p* = 0.034 *p* = 0.0036	
		50 μM + 20 μM NF Aβ 50 μM + 20 μM F Aβ	*p* = 0.0026 *p* = 0.0338	
		100 μM + 20 μM NF Aβ 100 μM + 20 μM F Aβ	*p* = 0.0002 *p* = 0.0066	
		200 μM + 20 μM NF Aβ 200 μM + 20 μM F Aβ	*p* = 0.0012 *p* > 0.05	

## Data Availability

Not applicable.
